# Generalization of the Ewens sampling formula to arbitrary fitness landscapes

**DOI:** 10.1371/journal.pone.0190186

**Published:** 2018-01-11

**Authors:** Pavel Khromov, Constantin D. Malliaris, Alexandre V. Morozov

**Affiliations:** Department of Physics and Astronomy and Center for Quantitative Biology, Rutgers University, Piscataway, New Jersey, United States of America; Fred Hutchinson Cancer Research Center, UNITED STATES

## Abstract

In considering evolution of transcribed regions, regulatory sequences, and other genomic loci, we are often faced with a situation in which the number of allelic states greatly exceeds the size of the population. In this limit, the population eventually adopts a steady state characterized by mutation-selection-drift balance. Although new alleles continue to be explored through mutation, the statistics of the population, and in particular the probabilities of seeing specific allelic configurations in samples taken from the population, do not change with time. In the absence of selection, the probabilities of allelic configurations are given by the Ewens sampling formula, widely used in population genetics to detect deviations from neutrality. Here we develop an extension of this formula to arbitrary fitness distributions. Although our approach is general, we focus on the class of fitness landscapes, inspired by recent high-throughput genotype-phenotype maps, in which alleles can be in several distinct phenotypic states. This class of landscapes yields sampling probabilities that are computationally more tractable and can form a basis for inference of selection signatures from genomic data. Using an efficient numerical implementation of the sampling probabilities, we demonstrate that, for a sizable range of mutation rates and selection coefficients, the steady-state allelic diversity is not neutral. Therefore, it may be used to infer selection coefficients, as well as other evolutionary parameters from population data. We also carry out numerical simulations to challenge various approximations involved in deriving our sampling formulas, such as the infinite-allele limit and the “full connectivity” assumption inherent in the Ewens theory, in which each allele can mutate into any other allele. We find that, at least for the specific numerical examples studied, our theory remains sufficiently accurate even if these assumptions are relaxed. Thus our framework establishes both theoretical and practical foundations for inferring selection signatures from population-level genomic sequence samples.

## Introduction

With the advent of high-throughput molecular biology techniques, it has recently become possible to carry out large-scale genotype-phenotype assays in molecular systems [1–5]. For example, Podgornaia and Laub have recently mapped all 20^4^ = 1.6 × 10^5^ possible combinations of four key residues in the *E. coli* protein kinase PhoQ, and assayed each mutant for the signaling function mediated by its binding partner PhoP [1]. This study revealed 1659 functional PhoQ variants, which can be thought of as forming the upper plane on the fitness landscape; all non-functional variants form the lower plane. The upper plane is divided into several clusters under single-point amino acid or nucleotide mutations—sequences within each cluster can mutate into each other through neutral substitutions only. The two-plane landscape is epistatic—the effect of a given mutation depends on the amino acids at the other three positions, in agreement with previous reports on the major role of epistasis in molecular evolution [6–9].

The picture of a “coarse-grained” fitness landscape stratified into several distinct phenotypes is in agreement with other recent high-throughput experiments aimed at elucidating the relationship between sequence and function [2–4, 7, 10, 11]. Although these experiments typically yield continuous distributions of selection coefficients, the distributions tend to be bi-modal, with one peak corresponding to strongly deleterious and lethal mutations and another to weakly deleterious and neutral ones [12–14]. These observations suggest stratifying the fitness landscape into functional and non-functional phenotypes; intermediate fitness states such as those corresponding to weakly deleterious phenotypes can be added if necessary to refine the picture.

Overall, given the astronomically large number of alleles, the typical size of neutrally-connected clusters of sequences can be assumed to be much larger than the population size. Then evolutionary dynamics on a multiple-plane landscape will be characterized by mutation-selection-drift balance [15–22] in the infinite-allele limit. At steady state, population statistics, such as the mean and the variance of the number of distinct alleles or the probability of observing a given pattern of allelic diversity in a sample of sequences, do not change anymore, even though the population continues to explore new alleles through mutation [22]. In the absence of selection, the steady-state allele sampling probability was derived by Ewens [23]. The Ewens sampling formula can be used to understand allelic diversity in neutral populations and to test for deviations from the neutral expectation; [24] its essential limitation is that, essentially, each allele is allowed to mutate into every other allele [22]. The Ewens formula arises naturally in many sampling problems in biological and physical sciences [25–27]. However, in order to understand molecular evolution in the presence of selection and make quantitative predictions of selection coefficients, it is necessary to extend it to more general fitness distributions.

Previous work in this area has focused mostly on the symmetric overdominance model, first analyzed in this context by Watterson [18, 28]. This is a diploid model in which all heterozygotes have the same selective advantage over all homozygotes, such that the mean population fitness depends on the square of allele frequencies. Since the sampling formula for this model is challenging to evaluate and therefore has never been used in practical calculations, subsequent work in the field focused on various approximations to the exact result, which require additional assumptions such as weak selection [18] or large sample sizes [29]. In particular, Joyce and collaborators have discussed asymptotic properties of the sampling distributions under a model of selection with multiple fitness states [30, 31], as well as the symmetric overdominance model [32]. More recently, Watterson’s model of selection was generalized by Handa [33] and Huillet [34], who considered mean population fitness involving allele frequencies raised to the arbitrary power *q* ≥ 1. They obtained sampling probabilities expressed in terms of multi-dimensional integrals which would be difficult to employ in practical calculations. In any event, only the *q* = 1 (neutral evolution) and *q* = 2 (symmetric overdominance) cases appear to have biological meaning.

Furthermore, Ethier and Kurtz have studied allelic diversity in a general model of selection in which fitness of each new allele is a symmetric function of the allelic states of its two parents, focusing on the proofs of existence and uniqueness of a steady state in the infinite-allele limit. [35, 36] Desai et al. have investigated sampling probabilities in a model (previously introduced by Charlesworth et al. [37] and Hudson and Kaplan [38]) based on a sequence of neutral and negatively selected sites [39]. This model has no interactions between sites, and therefore can be treated using the Poisson Random Field approach [40]. Since molecular evolution is characterized by prominent epistasis and correlated fitness values between parents and their offspring, the approach of Desai et al. cannot be applied to genomic data without careful numerical analysis of all the approximations involved. Finally, several prior publications have focused on steady-state population statistics other than sampling probabilities. In particular, Li used the steady-state approach to obtain the frequency spectrum for a general landscape, and derived expressions for the mean number of alleles in a sample, as well as the mean and the variance of heterozygosity [19–21]. Ewens and Li derived frequency spectra for landscapes with two and three distinct fitness states and used them to compute the mean number of distinct alleles and the mean heterozygosity [41]. Griffiths derived a general integral expression for the frequency spectrum in a genic selection model [42].

Here we demonstrate an extension of the Ewens sampling formula to arbitrary fitness landscapes with genic selection. First, we follow previous work [15–22] in assuming that the population adopts a steady state characterized by mutation-selection-drift balance. The steady state depends on the mean population fitness, which involves a linear combination of gene frequencies. Next, we derive a general sampling formula valid for any mutation rate *μ*, population size *N*, sample size *n* ≪ *N*, and the number of alleles *K* with arbitrary fitness. We find that the most general sampling formula is difficult to employ in numerical calculations with large finite values of *K*, but small values of *K* and the infinite *K* limit are more manageable. Here we focus on the infinite-allele (*K* → ∞) approximation with several phenotypic states, inspired by recent high-throughput molecular evolution experiments [1–5, 7, 10, 11]. We have developed a numerical technique based on the efficient calculation of Bell polynomials, which is distinct from previous efforts to compute sampling probabilities [43, 44]. Our approach enables us to study selection signatures and deviations from neutrality on landscapes with arbitrary fitness distributions.

We contrast our predictions with the effective population size approximation [37, 39]. We also compare our results with explicit simulations, using the Moran population genetics model [45] with single-point mutations as a benchmark against which the accuracy of the “full connectivity” assumption is checked. Finally, we investigate the limitations of the infinite-allele assumption. Our results are applicable to understanding the nature of allelic diversity under selection, mutation and drift. Moreover, our sampling formulas can form a basis of a quantitative, numerically feasible test for detecting the presence of selection and estimating its strength in evolving populations. Population-level allele diversity data are made increasingly available through high-throughput sequencing techniques, making our approach a practical and timely tool for studying the role of selection in evolution—a topic of much current interest and debate [14, 46–51].

## Results

### Sampling probability with selection

We consider a haploid population of fixed size *N* (our results also hold for diploid populations, as long as fitness values are assigned to individual genes rather than organisms). Each organism in the population is represented by a single allele in the state *i*, with fitness *f*_*i*_; there are *K* distinct allelic states. Mutations occur with a probability *μ* per generation, changing the original allele into one of the *K* − 1 remaining alleles. Thus the probability of offspring *A*_*j*_ produced by parent *A*_*i* ≠ *j*_ is *μ*/(*K* − 1) (note that our approach can be easily generalized to the case of final-state-dependent mutation rates: *μ*_*ij*_ = *μ*_*j*_, ∀*i* in *A*_*i*_ → *A*_*j*_). We can view this system as an “allelic network” with the topology of a complete graph, with *K* vertices representing allelic states and edges representing mutational moves. Stochastic evolution of the population can then be described using Moran [45, 52] or Wright-Fisher [15, 52] models of population dynamics.

The steady-state distribution of allelic frequencies for these models is given by [15–19]

$$\begin{array}{c} p\left(x\right)=\frac{1}{Z}e^{N\langle f\rangle }\prod_{i=1}^{K}x_{i}^{\epsilon -1}, \end{array}$$ (1)

where **x** = (*x*_1_, …, *x*_*K*_) is a vector of allelic frequencies, *ϵ* = *θ*/(*K* − 1) with *θ* = *Nμ* for Moran and *θ* = 2*Nμ* for Write-Fisher models correspondingly, $\langle f\rangle =\sum_{i=1}^{K}f_{i}x_{i}$ is mean population fitness, and *Z* is a normalization constant.

In many situations relevant to molecular evolution, the number of alleles *K* is much larger than the population size *N*. In this case, the steady state in terms of allele frequencies is unlikely to be reached on relevant evolutionary time scales. Mathematically, the *K* → ∞ limit of Eq 1 becomes ill-defined [53, 54]. Nonetheless, the steady state is well-defined in terms of allelic *counts* rather than frequencies of specific alleles [22]. In other words, the allelic diversity of the population (e.g. as characterized by the mean and the variance of the distribution of the number of distinct allelic types) is tractable and will no longer change in steady state, although new alleles will continue to be explored through mutation.

Since only a subset of the entire population is typically available for analysis, we shall focus on the probabilities of allelic counts in samples of size *n* ≪ *N*. To introduce the concept of allelic counts, let us for a moment consider a finite number of allelic types, e.g. *K* = 5, and call the corresponding alleles *A*, *B*, *C*, *D*, *E*. Suppose that we take a sample of *n* = 4 alleles from the population and we first observe allele *A*, then *C*, then *A* again, and finally *D*. We can record this sequence of alleles as an ordered list (*A*, *C*, *A*, *D*). However, typically we are not interested in the order in which alleles appear in the sample, and therefore record the result as an unordered list {*A*, *A*, *C*, *D*}, which shows that allele *A* has appeared twice and alleles *C* and *D* have appeared once each. Here we have used the notation {*a*, *b*, …, *z*} for unordered lists ({*a*, *b*, …, *z*} = {*b*, *a*, …, *z*}), and (*a*, *b*, …, *z*) for ordered lists ((*a*, *b*, …, *z*) ≠ (*b*, *a*, …, *z*)).

Alternatively, we can record non-zero allelic counts, which yields *n*_*A*_ = 2, *n*_*C*_ = 1, *n*_*D*_ = 1. Finally, we can dispense with the allele labels altogether, identifying each allele in the sample as either new or already seen. In this case, we are left with an unordered list of counts {2, 1, 1}, meaning that we have observed 4 alleles of 3 different types, with one type represented by two alleles and the other two types by one each. In general, we will refer to **n** = {*n*_1_, …, *n*_*k*_} as the sample configuration or the allelic counts. An equivalent representation would be to use a histogram which records how many groups of *j* identical alleles occur in the sample, with *j* ranging from 1 to *n*. In our example, there is one group of two identical alleles and two groups of one allele each, so that (*A*, *C*, *A*, *D*) is recorded as the allelic histogram (*a*_1_ = 2, *a*_2_ = 1, *a*_3_ = 0, *a*_4_ = 0). All results in the paper are presented in terms of the counts {*n*_1_, …, *n*_*k*_} rather than the histogram (*a*_1_, …, *a*_*n*_).

It turns out that the allelic counts are appropriate variables in the infinite allele limit. The celebrated Ewens sampling formula [22, 23] expresses the probability of observing a particular sample configuration **n** in the absence of selection:

$$\begin{array}{c} P\left[n\right]=N_{P}\frac{1}{k!}\frac{n!}{\prod_{i=1}^{k}n_{i}}\frac{\theta^{k}}{\theta^{\left(n\right)}}. \end{array}$$ (2)

where *N*_*P*_ is the total number of distinct permutations of the allelic counts, and *θ*^(*n*)^ = *θ*(*θ* + 1)…(*θ* + *n* − 1) is the rising factorial.

Following an approach developed by Watterson [18], we generalize the Ewens sampling formula to the case of multiple fitness states. We define ***γ***, a vector whose components, *γ*_*m*_, are fractions of all alleles with fitness *f*_*m*_. Allowing *m* to range from 1 to *M* ($\sum_{m=1}^{M}\gamma_{m}=1$) results in a landscape with *M* ≪ *K* distinct fitness states. Unless *γ*_*m*_ ∼ 1/*K*, there is an infinite number of alleles with the same fitness, so that the landscape looks like *M* fitness planes interconnected through mutations. For this reason we shall often refer to phenotypic states as fitness planes and to the fitness landscape as the multiple-plane landscape.

Our main result is the following expression for the sampling probability (details of the derivation are available in Materials and Methods):

P[n]=n!k!1∏i=1kniθkθ(n)×∑ν∈P(n)∑Y∈Y(n)F(γθ+νY;θ+n;β)F(γθ;θ;β)(ki1…iM)γ1i1…γMiM. (3)

**Fig 1:**
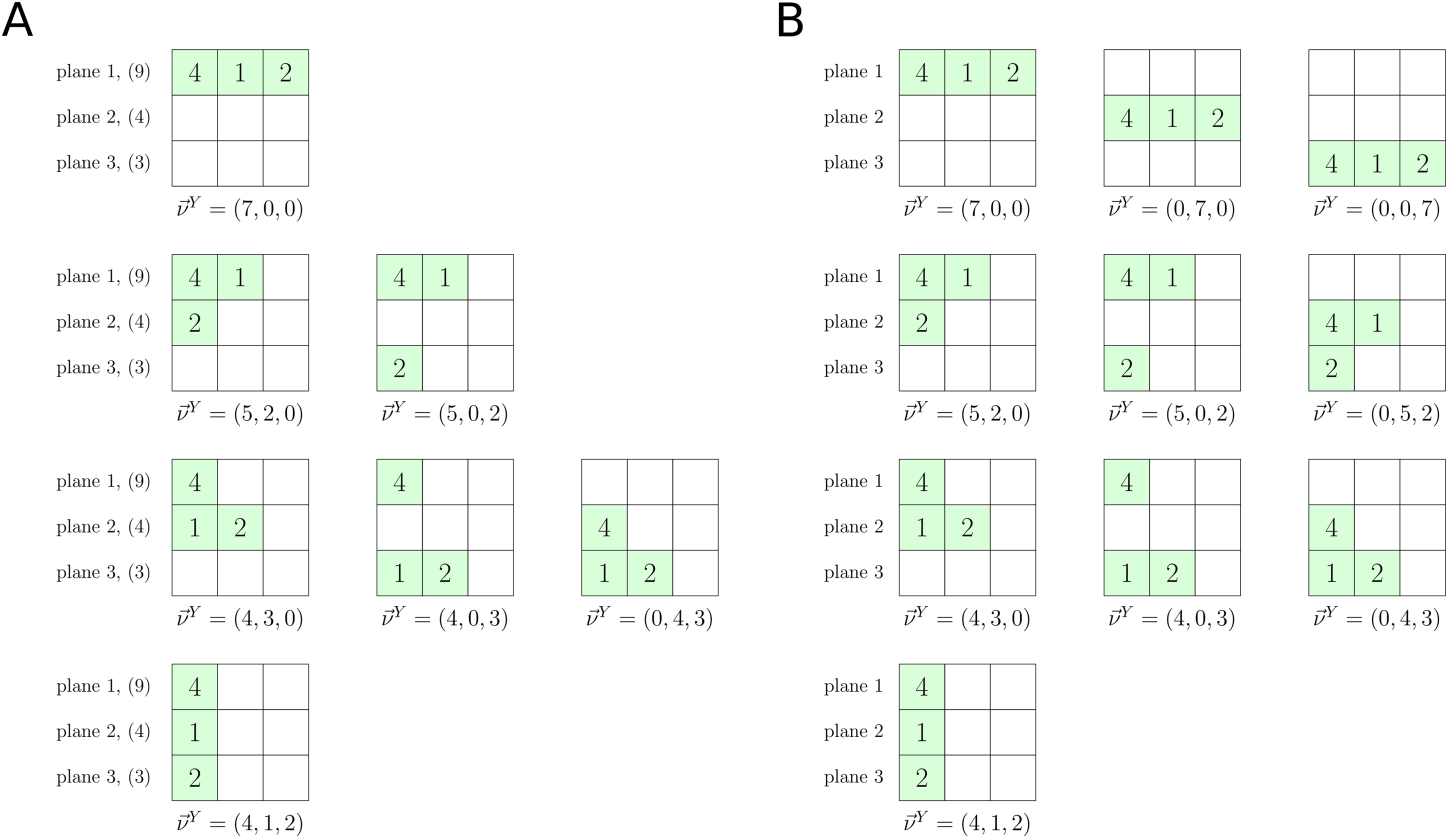
Summations in the sampling formula for a population with multiple fitness states. Illustration of summations over Y(I,n) and Y(n) in Eqs 31 and 32 respectively, for a list of allelic counts **n** = {4, 1, 2}. (A) The finite plane case. Finite plane capacities are shown in parentheses. (B) The infinite plane case.

Here, F(a;b;z) is a generalization of the confluent hypergeometric function ${}_{1}F_{1}\left(a;b;z\right)$ to vector arguments. The double sum in Eq 3 takes into account all possible ways of assigning observed allelic counts **n** to *M* fitness planes; ***ν***^*Y*^ is an auxiliary vector which encodes these assignments (see Materials and Methods and Fig 1 for extended explanations). Each assignment contributes differently to the final expression due to the non-trivial fitness landscape. The fitness values are stored in the vector ***β***, whose components are fitness differences *β*_*m*_ = *N*(*f*_*m*_ − *f*_1_) scaled by the population size *N*. For example, in the case of two fitness states *β*_1_ = 0 and *β*_2_ = *N*(*f*_2_ − *f*_1_) = *Ns*, where *s* is the selection coefficient. Finally, *i*_1_…*i*_*M*_ indicate the number of distinct allelic types sampled from the corresponding fitness plane ($\sum_{m=1}^{M}i_{m}=k$).

The first line in Eq 3 is simply the Ewens formula (Eq 2) without *N*_*P*_, which is the value returned by the double sum on the second line when all fitness values are equal. The version of the sampling formula with selection (Eq 3) suitable for a finite number of alleles *K* is provided in Materials and Methods. In the main text we shall focus on the infinite allele limit. Despite the seemingly complicated structure of Eq 3, it can be used in efficient numerical calculations. The following sections are devoted to exploring the properties of this formula and discussing its applicability and accuracy if some of the model assumptions are relaxed.

### The effective population size approximation

According to the effective population size (EPS) approximation [37, 39] in the monomorphic limit population dynamics is effectively neutral with a rescaled population size N*. Indeed, in this limit Eq 3 reduces to

$$\begin{array}{c} P\left[n\right]\underset{\theta \to 0}{\to }\frac{N_{P}}{k!}\frac{n!}{\prod_{i=1}^{k}n_{i}}\theta^{k-1}{\left(1-\gamma \right)}^{k-1} \end{array}$$ (4)

in the two-plane case. The *θ* → 0 limit corresponds to the *s* ≫ *μ* regime with *s* being finite; Eq 4 is the same as the neutral sampling formula (Eq 2) in the monomorphic limit if the population size is rescaled: *N* → N* = (1 − *γ*)*N*. This result can be generalized to the landscape with multiple fitness planes, in which case N* = *γ*_*m*_*N*, where *γ*_*m*_ is a fraction of nodes with the highest fitness.

However, the EPS approximation breaks down in the polymorphic regime. Indeed, if we take the *θ* → ∞ limit while keeping *s*/*μ* finite, it can be shown for the two-plane landscape that

$$\begin{array}{c} \frac{\mathbb{P}\left[n\right]}{\mathbb{P}\left[n,s=0\right]}\underset{\theta \to \infty }{\to }\sum_{m=0}^{\infty }c_{m}{\left(\frac{s}{\mu }\right)}^{m}\equiv \lambda \end{array}$$ (5)

where P[n,s=0] is given by Eq 2, and the coefficients *c*_*m*_ depend solely on the allelic counts *n*_1_, …, *n*_*k*_. Since the right-hand side of Eq 5 does not depend on the population size, it can be used to define N* = λ^1/(*k* − *n*)^ *N*. However, this definition will be sample-specific, as λ depends on the allelic counts via *c*_*m*_’s. Thus there is no universal rescaling of the population size in the strongly polymorphic regime, and therefore evolutionary dynamics is non-neutral.

### Detection of selection signatures

As discussed above, in general we expect allele diversity to deviate from neutrality, making it possible to detect selection signatures using a set of sequences sampled from the population. To investigate non-neutral population dynamics, we compute probabilities for all integer partitions **n** = {*n*_1_, …, *n*_*k*_} of *n* alleles sampled from the population evolving under selection (Eq 3), and compare them with steady-state partition probabilities obtained under neutral evolution (Eq 2) and the monomorphic EPS approximation (Eq 4).

**Fig 2:**
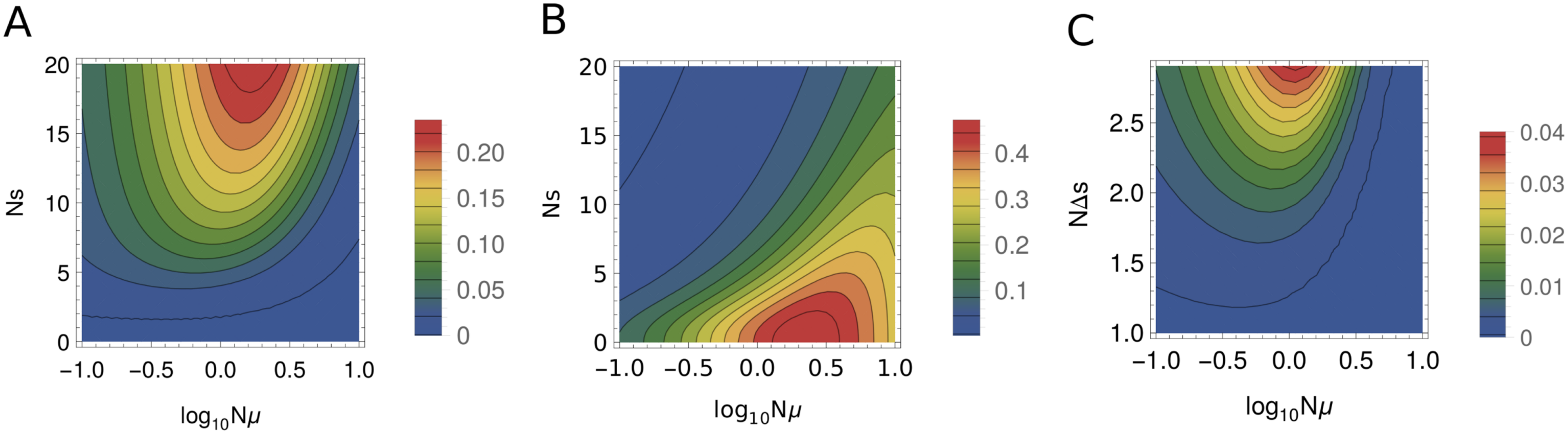
KL divergences of partition probabilities. Probabilities of all possible partitions of *n* = 3 alleles ({3}, {2, 1}, {1, 1, 1}) were sampled from a population of size *N* = 10^3^. (A) and (B) KL divergences for the two-plane fitness landscape as a function of the mutation rate *Nμ* and the selection coefficient *Ns* scaled by the population size, for partition probabilities with and without selection (A), and partition probabilities with selection compared with the EPS approximation (Eq 4) (B). (C) KL divergences for the sampling probabilities of all possible partitions on a three-plane vs. two-plane landscape. Alleles in the three planes have fitnesses 1, 1 + *s* − Δ*s* and 1 + *s* − Δ*s* respectively, with *Ns* = 6 for both two and three-plane landscapes.

We use the Kullback-Leibler (KL) distance to quantify the difference between two probability distributions [55]: *KL*(*p*||*q*) = ∑_*i*_ *p*_*i*_ log(*p*_*i*_/*q*_*i*_), where *i* is the partition label. For the two-plane system, we first compare partition probabilities under selection, $p_{i}=P\left[n,\theta ,\beta \right]$, with the corresponding neutral probabilities, $q_{i}=P\left[n,\theta ,\beta =0\right]$. In Fig 2A, we plot the KL divergence as a function of the mutation rate and the selection strength for the two-plane fitness landscape. We observe that evolutionary dynamics is essentially neutral if selection is weak (*s* ≤ *μ*); in addition, the range of selection coefficients for which neutrality holds increases in the monomorphic regime (*Nμ* ≤ 1). On the other hand, population statistics is clearly non-neutral when the population is polymorphic and when the separation between the two fitness planes is large. Next, we compute the KL divergence *KL*(*p*||q*) between the EPS probability distribution, $q_{i}^{*}=P\left[n,\theta^{*},\beta =0\right]$, where θ* = (1 − *γ*)*θ*, and *p*_*i*_ (Fig 2B). We see that the EPS approximation fails in the polymorphic, weak-selection regime. Overall, the neutral and EPS approximations are approximately complementary: for example, in the strong-selection (*s* ≫ *μ*) polymorphic regime, when evolutionary dynamics becomes non-neutral, it is well approximated by the EPS model.

In Fig 2C we show KL divergences between partition probability distributions on two- and three-plane fitness landscapes. We observe that the partition probabilities are essentially two-plane (i.e., there are no selection signatures indicating presence of intermediate-fitness alleles) if the population is monomorphic (*Nμ* ≤ 1), or if the distance between the two upper planes is smaller than the mutation rate (Δ*s* ≤ *μ*). However, there is a considerable parameter region in which deviations between two and three-plane sampling probabilities appear to be significant (with KL divergences between the two distributions of 0.01 or more), making it possible to detect three distinct fitness states in the sampling data.

### Mutation load

By definition, the mutation load is given by [52, 56] *L* = (*f*_max_ − 〈 *f* 〉)/*f*_max_, where *f*_max_ is the maximum fitness and $\langle f\rangle =\sum_{i=1}^{K}x_{i}f_{i}$ is the mean population fitness. To estimate the mutation load at steady state, we compute the expected value of the mean population fitness over multiple realizations of the stochastic process, E[〈f〉].

For the two-plane system, this computation leads to

L=sγ1+s(1F1(γθ+1;θ+1;-Ns)(1F1(γθ;θ;-Ns). (6)

Another indicative quantity is the average fraction of the population with low fitness, $E[x_{\text{low}}]$. For the two-plane system it is given by $E\left[x_{\text{low}}\right]=L\left(1+s\right)/s.$

**Fig 3:**
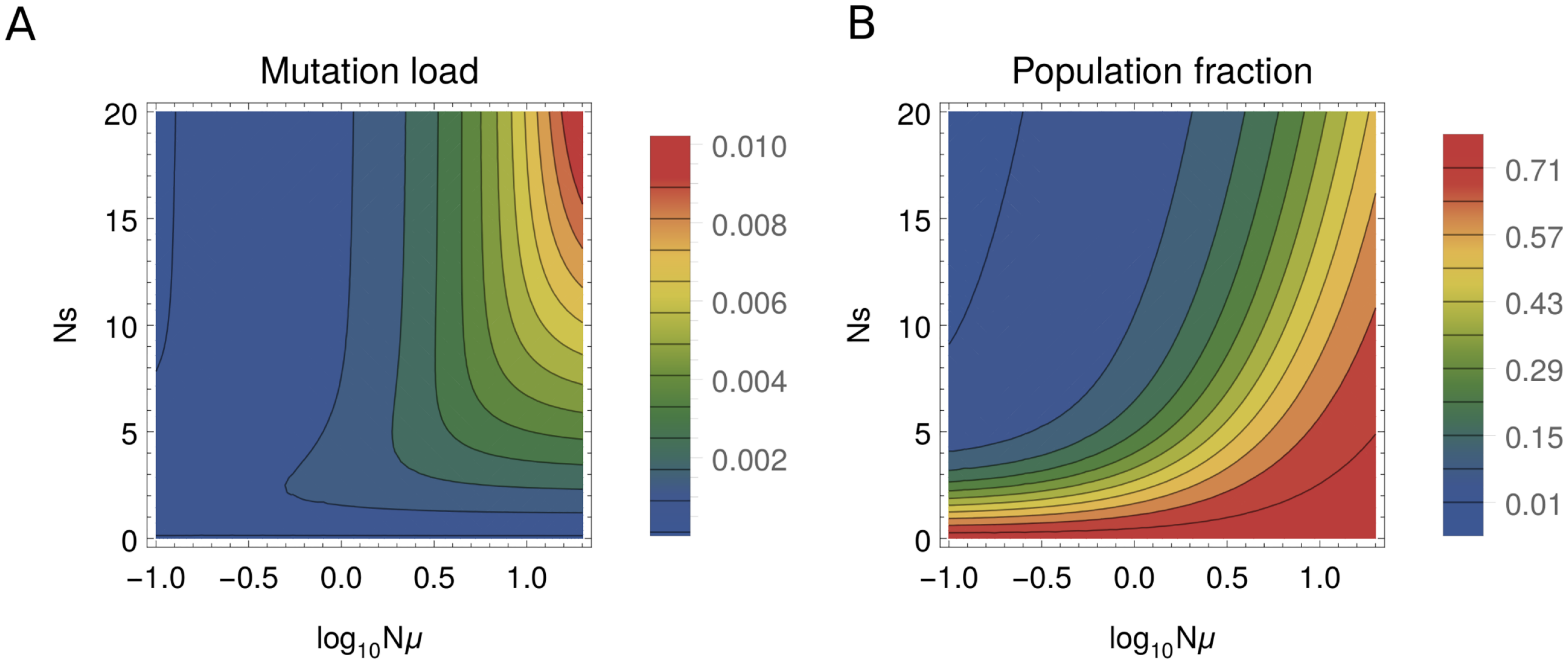
Mutation load and population fraction for the two-plane fitness landscape. (A) Mutation load (Eq 6) and (B) population fraction in the lower plane, as a function of the mutation rate (*Nμ*) and the selection strength (*Ns*) rescaled by the population size.

Values of mutation load for the two-plane fitness landscape are shown in Fig 3A over a range of selection strengths and mutation rates. As expected, we observe that the largest deviations from the maximum fitness occur in the strong-mutation, strong-selection regime, where a fraction of the population is constantly displaced to the lower plane by mutation, incurring a fitness cost. Correspondingly, at a given value of selection strength the mutation load increases with the mutation rate. In the monomorphic regime the mutation load is vanishingly low because the entire population condenses to a single allelic state and moves randomly on the upper plane. The fraction of the population on the lower fitness plane is shown in Fig 3B. The fraction is high when the separation between the two planes is low and, at a fixed separation, it increases with the mutation rate.

### Fitness landscape models and numerical simulations

To check our main result (Eq 3), we have compared it to the outcomes of numerical simulations of two models. In the first model, each allele is allowed to mutate into any of the other *K* − 1 alleles with equal probability. We call this model fully-connected (FC); derivations of the Ewens sampling formula and our generalization of it (Eq 3) were carried out for the FC model. The second model is more realistic: an organism is represented by a sequence of integers *S* = (*a*_1_, …, *a*_*L*_) of length *L* and alphabet size *A*, meaning that 0 ≤ *a*_*i*_ ≤ *A* − 1. A mutation replaces an integer at a randomly chosen site with one of the remaining *A* − 1 integers; all the replacements have equal probabilities. We call this model a single-point mutation (SPM) model; it is a more realistic description of protein or nucleotide sequence evolution.

To assign a fitness value to each allele, we focus on the landscapes in which alleles can have either low or high fitness values (the two-plane model), or low, intermediate, and high fitness values (the three-plane model). The fractions of alleles found in each plane are given by ***γ***: ***γ*** = (*γ*, 1 − *γ*) for the two-plane model and ***γ*** = (*γ*_1_, *γ*_2_, 1 − *γ*_1_ − *γ*_2_) for the three-plane model. In the FC model, the mutational neighborhood of each allele is the same, so that any desired allele fractions ***γ*** can be implemented. However, in the SPM model the fractions of neutral, beneficial and deleterious moves in each plane will depend on ***γ*** and the assignment of states to planes. We wished to produce non-trivial distributions of neutral moves on the fitness planes, with mutational neighborhoods of some alleles being completely neutral in each plane. Another condition was that the number of alleles in each plane should decrease with its fitness, to reflect the fact that beneficial mutations are rare.

To fulfill these requirements, we chose to assign fitness values in the SPM model in the following way. We use the sequence length *L* = 10 and the alphabet size *A* = 4. For each sequence *S* = (*a*_1_, …, *a*_*L*_) we compute a score *z* = *a*_1_ + … + *a*_*L*_. We compare these scores with a set of cutoffs (*c*_1_, …, *c*_*M*−1_) for the *M*-plane landscape. For the two-plane landscape, the fitness is 1 if *z* ≤ *c*_1_, and 1 + *s* otherwise. We use the cutoff *c*_1_ = 17, which yields ***γ*** = (0.758, 0.242). For the three-plane landscape, if *z* ≤ *c*_1_ the fitness is 1, if *c*_1_ < *z* ≤ *c*_2_ the fitness is 1 + *s* − Δ*s*, and if *z* > *c*_2_ the fitness is 1 + *s* + Δ*s*. We choose the cutoffs *c*_1_ = 17 and *c*_2_ = 21, which lead to ***γ*** = (0.758, 0.210, 0.032). In order to compare FC and SPM simulations directly, we use the same values of ***γ*** in the corresponding FC models.

Our numerical simulations have been carried out using the Moran model of population genetics [22, 45]. Specifically, we have evolved a population of *N* = 10^3^ haploid organisms, each of which could be in one of *K* allelic states. At each step a parent is chosen by randomly sampling the population with weights proportional to the fitness of each individual. An offspring is then produced as an exact copy of the parent. Next, the offspring undergoes mutation with the probability *μ*. Finally, the population is uniformly sampled to choose an organism that will be replaced by the offspring, keeping the overall population size constant. Probabilities of sampling *n* individuals from the population were calculated as averages over 10^6^ samples gathered from 10^3^ independent runs. For each run, a randomly generated initial population was evolved to steady state, after which *n* individuals were sampled from the population with replacement 10^3^ times, waiting ∼1/*μ* generations between subsequent samples.

Note that in the neutral case the exact mapping between *θ* and *μ* is given by *θ* = *Nμ*/(1 − *μ*) for the Moran model. [22] However, it is unclear if this mapping can be extended to the non-neutral cases considered here. In any event, for the population size and the values of *θ* investigated below, *μ* = *θ*/(*N* + *θ*) ≃ *θ*/*N*. Therefore, we use the diffusion theory result *θ* = *Nμ* in comparing theoretical predictions with numerical simulations.

### Partition probabilities on fully-connected vs. single-point-mutant networks

**Fig 4:**
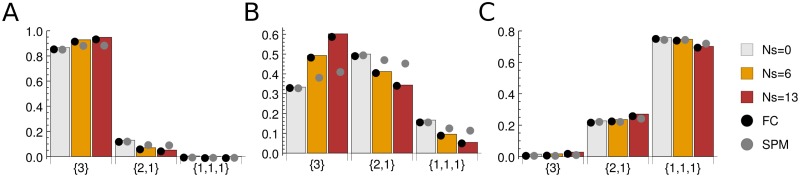
Partition probabilities for the two-plane fitness landscape. Shown are sampling probabilities of all partitions with *n* = 3: {3}, {2, 1}, {1, 1, 1}. Bars: theoretical predictions in the infinite allele limit. Black circles: numerical simulations on the FC sequence network. Grey circles: numerical simulations on the SPM sequence network. In all simulations, alphabet size *A* = 4, sequence length *L* = 10, and population size *N* = 10^3^ were used. Partition probabilities were estimated from 10^6^ samples as described in the main text. (A) Monomorphic population, *Nμ* = 0.1. (B) Weakly polymorphic population, *Nμ* = 1.0. (C) Strongly polymorphic population, *Nμ* = 10.0. The corresponding KL divergences are listed in Table 1. Note that the error bars of the partition probabilities are too small to be shown, due to extensive sampling in our numerical simulations.

Here we investigate the extent to which sampling probabilities change in the SPM sequence evolution model described above, compared to the FC fitness landscape. We are especially interested in the limits of the predictive power of our theoretical framework, which necessarily involves the FC assumption. In Fig 4 and Table 1 we compare theoretical predictions with numerical simulations on the FC and SPM networks in the two-plane system for the sample of *n* = 3 alleles. Overall, as expected, we observe an excellent agreement between theory and simulations on FC networks. Furthermore, we see that the agreement between SPM simulations and our theoretical results is reasonable: in nearly all cases, the predicted ranking of the sample partitions, as well as the ranking of any given sample partition with respect to the selection strength, *Ns*, are preserved. The largest discrepancies occur in the weakly polymorphic (*Nμ* = 1), non-neutral regime (*Ns* = 6, 13).

**Table 1:** KL divergences between theoretical predictions and numerical simulations for single-plane, two-plane (Fig 4), and three-plane (Fig 5) fitness landscapes, with the sample size *n* = 3.

		Single-plane landscape	Two-plane landscape		Three-plane landscape	
		*Ns* = 0	*Ns* = 6	*Ns* = 13	*Ns* = 6 ± 3	*Ns* = 13 ± 5
*Nμ* = 0.1	**FC**	1 × 10^−5^	2 × 10^−5^	3 × 10^−5^	4 × 10^−5^	2 × 10^−6^
	**SPM**	1 × 10^−5^	9 × 10^−3^	2 × 10^−2^	2 × 10^−2^	3 × 10^−2^
	**Ratio**	1.000	0.452	0.425	0.370	0.380
*Nμ* = 1.0	**FC**	2 × 10^−5^	8 × 10^−5^	1 × 10^−4^	1 × 10^−6^	6 × 10^−6^
	**SPM**	1 × 10^−4^	2 × 10^−2^	9 × 10^−2^	8 × 10^−2^	2 × 10^−1^
	**Ratio**	1.000	0.363	0.508	0.378	0.434
*Nμ* = 10.0	**FC**	1 × 10^−6^	6 × 10^−5^	2 × 10^−4^	2 × 10^−5^	4 × 10^−5^
	**SPM**	1 × 10^−4^	4 × 10^−5^	3 × 10^−3^	2 × 10^−4^	2 × 10^−2^
	**Ratio**	1.000	0.331	0.345	0.595	0.488

Note: **FC** = KL(*p* = numerical FC || *q* = theory), **SPM** = KL(*p* = numerical SPM || *q* = theory), **Ratio** = KL(*p* = theory || *q* = numerical SPM)/KL(*p* = theory || *q* = numerical neutral SPM).

**Fig 5:**
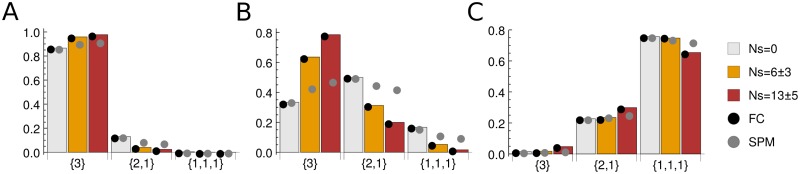
Partition probabilities for the three-plane fitness landscape. All notation and symbols are as in Fig 4. The corresponding KL divergences are listed in Table 1.

The situation is qualitatively similar when a three-plane fitness landscape is considered (Fig 5, Table 1). We again observe an excellent agreement between theory and FC simulations and, overall, a reasonable agreement between theory and SPM simulations, with the largest discrepancies again occurring in the weakly polymorphic, non-neutral regime. These observations remain true when samples with *n* = 4 and 5 alleles are considered (Tables 2 and 3).

**Table 2:** Same as Table 1, but for the sample size *n* = 4.

		Single-plane landscape	Two-plane landscape		Three-plane landscape	
		*Ns* = 0	*Ns* = 6	*Ns* = 13	*Ns* = 6 ± 3	*Ns* = 13 ± 5
*Nμ* = 0.1	**FC**	1 × 10^−5^	6 × 10^−6^	6 × 10^−6^	1 × 10^−6^	5 × 10^−6^
	**SPM**	1 × 10^−5^	9 × 10^−3^	2 × 10^−2^	2 × 10^−2^	4 × 10^−2^
	**Ratio**	1.000	0.394	0.527	0.397	0.432
*Nμ* = 1.0	**FC**	9 × 10^−5^	3 × 10^−5^	8 × 10^−5^	2 × 10^−4^	2 × 10^−4^
	**SPM**	9 × 10^−4^	3 × 10^−2^	1 × 10^−1^	1 × 10^−1^	3 × 10^−1^
	**Ratio**	1.000	0.527	0.542	0.442	0.486
*Nμ* = 10.0	**FC**	2 × 10^−5^	6 × 10^−6^	7 × 10^−5^	7 × 10^−6^	1 × 10^−5^
	**SPM**	2 × 10^−4^	1 × 10^−4^	3 × 10^−3^	2 × 10^−4^	2 × 10^−2^
	**Ratio**	1.000	0.418	0.199	0.677	0.406

**Table 3:** Same as Table 1, but for the sample size *n* = 5.

		Single-plane landscape	Two-plane landscape		Three-plane landscape	
		*Ns* = 0	*Ns* = 6	*Ns* = 13	*Ns* = 6 ± 3	*Ns* = 13 ± 5
*Nμ* = 0.1	**FC**	1 × 10^−5^	2 × 10^−5^	3 × 10^−5^	5 × 10^−6^	3 × 10^−6^
	**SPM**	3 × 10^−5^	1 × 10^−2^	2 × 10^−2^	3 × 10^−2^	4 × 10^−2^
	**Ratio**	1.000	0.441	0.385	0.379	0.429
*Nμ* = 1.0	**FC**	9 × 10^−5^	1 × 10^−4^	3 × 10^−4^	7 × 10^−4^	4 × 10^−5^
	**SPM**	5 × 10^−4^	4 × 10^−2^	1 × 10^−1^	1 × 10^−1^	3 × 10^−1^
	**Ratio**	1.000	0.428	0.485	0.426	0.514
*Nμ* = 10.0	**FC**	1 × 10^−5^	1 × 10^−5^	5 × 10^−4^	1 × 10^−4^	1 × 10^−3^
	**SPM**	1 × 10^−3^	5 × 10^−4^	8 × 10^−3^	4 × 10^−4^	4 × 10^−2^
	**Ratio**	1.000	0.461	0.548	0.546	0.516

Finally, we have checked whether our theoretical predictions, which rely on the full-connectivity assumption, are closer to the non-neutral rather than neutral SPM steady-state dynamics in numerical simulations: if this is the case, we should be able to predict selection signatures in populations evolving under single-point mutations using our methodology. We have computed the ratio of KL distances defined in the Table 1 caption; this ratio is less than 1 if the theoretical predictions with selection are closer to the corresponding SPM simulation than to the neutral SPM simulation, and greater than 1 otherwise. We observe that the ratio is less than 1 in all cases with selection and for all sample sizes (Tables 1–3), indicating that the error introduced by the FC assumption is less than the distance between selective and neutral systems (note that the ratio is 1 by definition in the single-plane neutral case).

### Infinite-allele assumption

**Fig 6:**
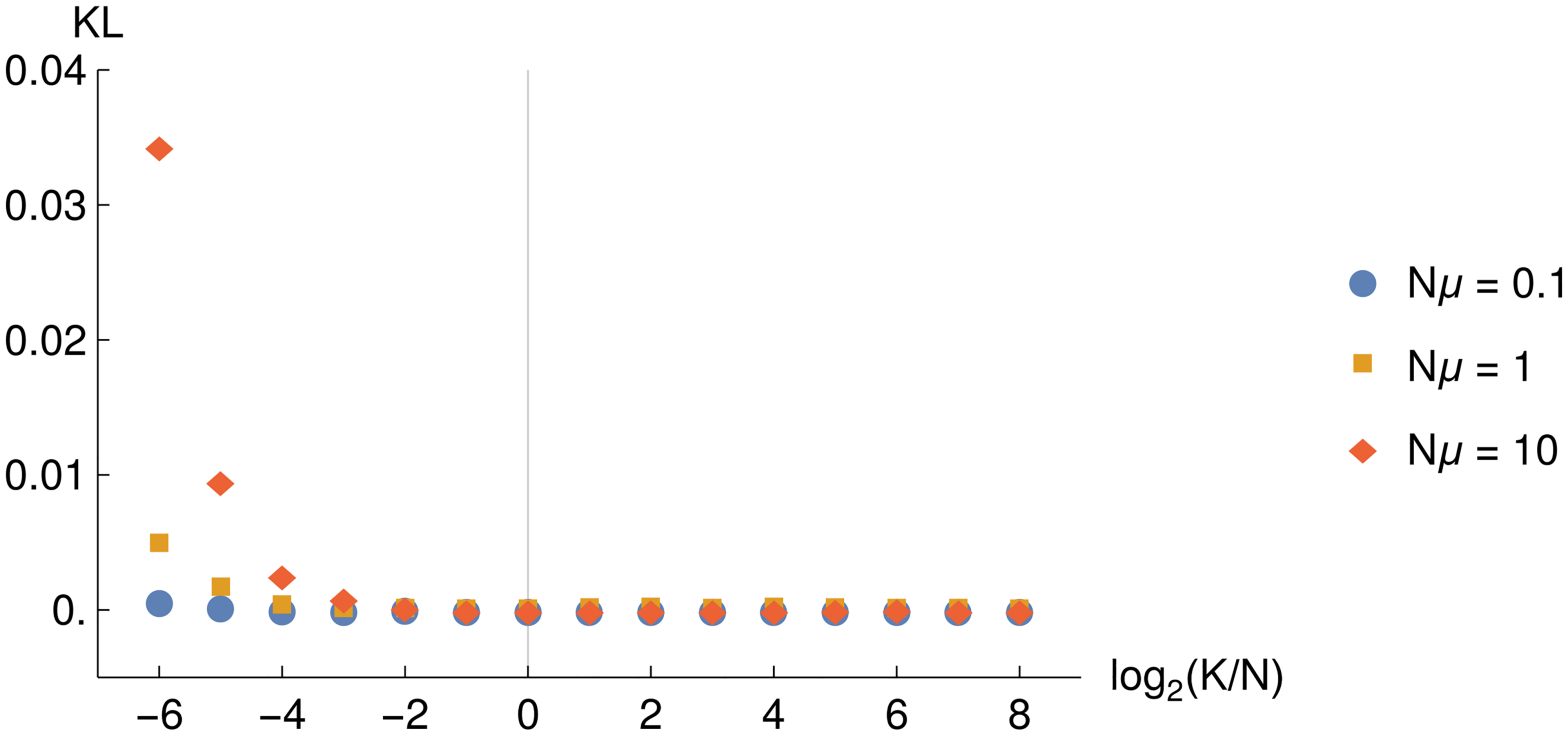
Test of the infinite-allele assumption. Shown are KL divergences between computational and theoretical partition probabilities on the FC two-plane fitness landscape (*Ns* = 6, ***γ*** = (0.758, 0.242)), as a function of the log ratio between the total number of alleles *K* and the population size *N*. The sample size is *n* = 3; partition probabilities were estimated from 10^6^ samples. Population size is *N* = 10^3^, and the total number of alleles is *K* = 10^3^ × 2^*i*^, *i* ∈ {−6…8}. For smaller networks, the number of sequences in the upper and lower planes had to be rounded to the nearest integer. Diamonds: polymorphic population (*Nμ* = 10.0), squares: weakly polymorphic population (*Nμ* = 1.0), circles: monomorphic population (*Nμ* = 0.1). The solid vertical line corresponds to *K* = *N*.

Although our approach is valid for an arbitrary number of alleles *K*, statistics of allele diversity in a population under selection become substantially easier to deal with in the infinite-allele limit. As discussed in the Introduction, this limit is justified since our focus here is on evolution of protein, RNA and DNA sequences, where the number of alleles grows exponentially with sequence length. Nonetheless, we have systematically investigated the extent of deviations between our infinite-allele theoretical results and simulations as the number of alleles *K* decreases and becomes comparable to the population size *N*. Fig 6 shows the KL divergence between partition probabilities derived theoretically for the two-plane landscape in the infinite-allele limit (Eq 3) and obtained numerically on finite-size FC networks. We consider three regimes: monomorphic (*Nμ* = 0.1), weakly polymorphic (*Nμ* = 1.0), and strongly polymorphic (*Nμ* = 10.0). In the latter two cases, noticeable deviations between theory and simulations begin to appear below the *K* ∼ *N* regime; the agreement improves as the population becomes more monomorphic. We conclude that our theory is applicable over a wide range of mutation rates, as long as the network size is comparable to, or greater than, the population size.

## Discussion and conclusion

One of the most challenging problems in evolutionary biology is to understand evolutionary dynamics of molecular loci, such as protein or RNA-coding sequences, or gene regulatory regions. The number of nucleotides at these loci, *L*, is large enough so that the total number of possible sequences, *K* = *A*^*L*^, is astronomical, far exceeding the population size *N*. Under these conditions the evolution of a molecular locus, assumed to be decoupled by recombination from the rest of the genome, reaches a “de-labelled” steady state. The allelic diversity in the steady-state population is determined by the balance of forces of selection and drift on one hand, and mutation on the other. The former act to reduce allelic diversity, while the latter acts to increase it. As a result, population statistics such as the mean number of distinct alleles, or the probability of seeing a certain allelic configuration in a sample, do not change with time, even though new genotypes continue to be explored on the effectively infinite allelic network.

The steady-state allelic diversity in an infinite-allele neutral system was explored by Ewens [22, 23]. The main result of that study, the Ewens sampling formula, is widely used in population genetics. However, selection is bound to play a key role in molecular evolution, and recent high-throughput studies connecting protein sequences with phenotypes [1–4, 7, 10, 11] reveal a more complex picture of molecular evolution: generally, a functional protein is disrupted by a fraction of mutations (e.g., through substitution of a hydrophobic residue for a hydrophilic one in the protein core). Other mutations do not significantly change protein stability, binding affinity, or binding specificity, and are therefore effectively neutral. Occasionally, a mutation is found which increases the fitness of an already functional, adapted protein, but these mutations are very infrequent. Overall, recent experimental studies indicate that “coarse-grained” fitness landscapes comprised of multiple interconnected planes (i.e., several distinct fitness states) are a reasonable representation. The simplest landscape of this kind has just two fitness states, with functional sequences on the upper plane and non-functional sequences on the lower plane [1]. Multiple-plane fitness landscapes constructed in this way are characterized by extensive epistasis under the single-point mutational move set, which is likely to be pervasive in molecular evolution [6–9].

Since molecular evolution may be described by steady-state dynamics on multiple-plane fitness landscapes, it is of great interest to generalize the Ewens sampling formula to arbitrary fitness distributions, and to the case of several distinct fitness states in particular. Tractable expressions for sampling probabilities would enable inference of selection coefficients, relative numbers of alleles in each fitness state, and mutation rates, using DNA, RNA, or protein sequences sampled from the population as input to the inference procedure. Here we report an extension of the Ewens sampling formula to arbitrary fitness distributions, focusing on the multiple-plane case which yields substantial simplifications in the infinite-allele limit. Unlike techniques based on the Poisson random field framework [40], such as the sampling probability formulas developed by Desai et al. [39], our approach does not rely on assuming independent evolution at each site along the sequence. However, an essential drawback of the Ewens sampling formula and our generalization of it is the “full-connectivity” assumption (i.e., that each allele can mutate into every other allele). Furthermore, the sampling formula becomes intractable for large sample sizes, since the number of terms to sum over in Eq 3 becomes too large.

Therefore, in order to study the limits of applicability of our theory, we have carried out extensive comparisons with numerical simulations on multiple-plane fitness landscapes. First, we checked the full-connectivity assumption inherent in the Ewens approach by comparing the sampling probabilities of our theory with those obtained by simulation of steady-state populations evolving on single-point-mutant networks. We find that the agreement, although dependent on the details of the fitness landscape model, the values of selection coefficients, and mutation rates (and least reliable in the weakly polymorphic regime), remains strong enough overall to encourage application of our theoretical results to sequence data. We also find that the error introduced by the full-connectivity assumption, as measured by the KL distance, is less than the distance between sampling probabilities in neutral and non-neutral systems. Note that our SPM model of the fitness landscape was constructed specifically to create a non-trivial distribution of neutral, deleterious and beneficial single-point mutations for the alleles, in some sense making it as distant from the fully connected network as possible. Thus we expect the errors inherent in our theoretical framework to be smaller (or at least not much worse) in applications to natural systems. Second, we have checked the infinite-allele assumption by systematically reducing the number of alleles until it became lower than the population size. We find that, for a wide range of mutation rates, deviations between theory and simulations become significant only when the number of alleles approaches the population size from above. Thus our assumption of the infinite network size is justified for sufficiently long loci, such as those encoding transcribed or regulatory regions.

Robust inference of selection coefficients from a sample of sequences collected from an evolving population requires statistics of allelic diversity to deviate substantially from the neutral expectation. If selection cannot be ruled out *a priori*, the use of our generalized Ewens sampling formula, which is valid throughout the entire parameter space, is necessary for inferring selection signatures and mutation rates from data. Moreover, allelic diversity generated by steady-state evolutionary dynamics on a three-plane fitness landscape is sufficiently distinct from its two-plane counterpart in the strong-selection, weakly polymorphic regime, opening up a possibility of inferring multiple selection coefficients from a sample of sequences. Another hallmark of non-neutral population dynamics is de-localization of the population to multiple fitness planes. With a two-plane landscape, we expect the fraction of the population on the lower plane to increase with the mutation rate and decrease with the distance between the two planes. Our investigation of the mutation load confirms these predictions.

In summary, we have generalized the Ewens sampling formula to populations evolving under selection. Although in principle our results are valid for arbitrary fitness distributions, focusing on the infinite allele limit and landscapes characterized by several distinct fitness states yields substantial simplifications, making our approach computationally tractable and thus applicable to inferring selection signatures from high-throughput sequence data. Such multiple-state “coarse-grained” fitness distributions appear to be a reasonable starting point supported by recent large-scale genotype-phenotype maps in molecular systems [1–4, 7, 10, 11]. Unlike previous approaches, we do not assume that each site along the sequence evolves independently—an assumption that has recently been challenged in molecular evolution studies [6–9]. However, we do make the infinite allele assumption, and, as in the Ewens original formula [23], assume that each allele can mutate into any other allele. Therefore, we check our theory against numerical simulations in model systems where these assumptions are relaxed, and find that our predictions remain accurate enough to enable inference of evolutionary parameters from sequencing data.

## Materials and methods

### Allele frequency distribution

Eq 1 can be rewritten as follows:

$$\begin{array}{c} p\left(x\right)=\frac{1}{B(\epsilon )F(\epsilon ;|\epsilon |;\beta )}\prod_{i=1}^{K}x_{i}^{\epsilon -1}e^{\beta_{i}x_{i}}, \end{array}$$ (7)

where ***ϵ*** = (*ϵ*, …, *ϵ*) is a *K*-dimensional vector of rescaled mutation rates, |***ϵ***| = *Kϵ* ≃ *θ* is the *L*_1_-norm of ***ϵ***,

$$\begin{array}{c} B\left(a\right)=\frac{\prod_{i=1}^{K}\Gamma \left(a_{i}\right)}{\Gamma (\sum_{i=1}^{K}a_{i})} \end{array}$$ (8)

is the generalized beta function, and

$$\begin{array}{c} F\left(a;b;z\right)=\sum_{j_{1}=0}^{\infty }\ldots \sum_{j_{K}=0}^{\infty }\frac{a_{1}^{\left(j_{1}\right)}\ldots a_{K}^{\left(j_{K}\right)}}{b^{\left(j_{1}+\ldots +j_{K}\right)}}\frac{z_{1}^{j_{1}}}{j_{1}!}\ldots \frac{z_{K}^{j_{K}}}{j_{K}!}=\sum_{j=0}^{\infty }\frac{B_{j}\left(\alpha_{1},\ldots ,\alpha_{j}\right)}{j!b^{\left(j\right)}} \end{array}$$ (9)

is a generalization of the confluent hypergeometric function ${}_{1}F_{1}\left(a;b;z\right)$ to vector arguments. Here, *a*^(*j*)^ = Γ(*a* + *j*)/Γ(*a*) is the rising factorial, *B*_*j*_ is the *j*th complete Bell polynomial, and $\alpha_{j}=\left(j-1\right)!\sum_{i=1}^{n}a_{i}z_{i}^{j}$. To obtain Eq 7, we have used the following result for integrating over the (*K* − 1)-dimensional simplex *Σ*_*K* − 1_:

$$\begin{array}{c} \int_{\Sigma_{K-1}}\prod_{i=1}^{K}x_{i}^{\nu_{i}-1}dx_{i}=\frac{\prod_{i=1}^{K}\Gamma \left(\nu_{i}\right)}{\Gamma (\sum_{i=1}^{K}\nu_{i})}. \end{array}$$ (10)

A (*K* − 1)-dimensional simplex *Σ*_*K* − 1_ is a subspace of $R^{K}:\left(x_{1},\ldots ,x_{K}\right)\in {\left[0,1\right]}^{K}$ which satisfies $\sum_{i=1}^{K}x_{i}=1$. We have expanded the exponent in Eq 1 in a Taylor series and applied Eq 10 to each term in the resulting expansion.

### Strongly monomorphic limit

In this limit the mutation rate tends to zero while the population size is kept fixed, *ϵ* → 0 [52, 56–58]. Consider the Fourier transform of the steady-state distribution in Eq 7:

$$\begin{array}{c} \tilde{p}\left(k\right)=\int_{\Sigma_{K-1}}dx\;e^{ik\cdot x}p\left(x\right), \end{array}$$ (11)

where the integral is over the (*K* − 1)-dimensional simplex. Using Eq 9, we can write the Fourier transform as a ratio of two generalized hypergeometric functions:

$$\begin{array}{c} \tilde{p}\left(k\right)=\frac{F(\epsilon ;|\epsilon |;\beta +ik)}{F(\epsilon ;|\epsilon |;\beta )}. \end{array}$$ (12)

Taking the *ϵ* → 0 limit yields

$$\begin{array}{c} {\tilde{p}}_{\text{mono}}\left(k\right)=\frac{\sum_{m=1}^{K}e^{\beta_{m}+ik_{m}}}{\sum_{m=1}^{K}e^{\beta_{m}}}. \end{array}$$ (13)

Thus the steady-state distribution in the monomorphic limit is given by:

$$\begin{array}{c} p_{\text{mono}}\left(x\right)=\int \frac{dx}{\text{Vol}(\Sigma_{K-1})}e^{-ik\cdot x}{\tilde{p}}_{\text{mono}}\left(k\right)=\frac{\sum_{m=1}^{K}e^{\beta_{m}}\delta \left(x-1_{m}\right)}{\sum_{m=1}^{K}e^{\beta_{m}}}, \end{array}$$ (14)

where $\text{Vol}\left(\Sigma_{K-1}\right)=\sqrt{K}/\left(K-1\right)!$ is the volume of the (*K* − 1)-dimensional simplex and (**1**_*m*_)_*i*_ = *δ*_*mi*_. The population resides in one of the *K* monomorphic states available to it, with the probability of being in a particular state exponentially weighted by its fitness [59–61].

### Probability of a sample of alleles

In this section we derive the sampling probability when the number of alleles *K* is finite. Let us find the probability P[n] of observing counts **n** = {*n*_1_, …, *n*_*k*_}, assuming that the population has reached steady state in terms of its allelic diversity. Before considering general case, we illustrate our approach using an example with only *K* = 3 allelic types: A=(A,B,C). We wish to calculate the probability of observing counts {2, 1} in a sample of size *n* = 3, which is assumed to be much less than the population size *N*. There are 18 samples that contribute to this counts:

$$\begin{array}{c} \begin{array}{ccc} AAB & ABA & BAA\\ AAC & ACA & CAA\\ BBC & BCB & CBB\\ ABB & BAB & BBA\\ ACC & CAC & CCA\\ BCC & CBC & CCB \end{array} \end{array}$$

The probability of choosing *A* first, then *A* again and finally *B* is

$$\begin{array}{c} \begin{array}{cc} P[(A,A,B)] & =\int x_{A}^{2}x_{B}^{1}\;p\left(x_{A},x_{B},x_{C}\right)\;dx_{A}dx_{B}dx_{C}\\ & =\int x_{A}^{2}x_{B}^{1}\;p\left(x_{A},x_{B}\right)\;dx_{A}dx_{B}, \end{array} \end{array}$$ (15)

where *p*(*x*_*A*_, *x*_*B*_, *x*_*C*_) is given by Eq 7. Consequently, the probability of observing two *A*’s and one *B* in *any* order is given by [18]

P[{A,A,B}]=(321)∫xA2xB1p(xA,xB)dxAdxB, (16)

where (321) is the multinomial coefficient. Introducing a set $S_{2}A=\left\{\left(A,B\right),\left(A,C\right),\left(B,C\right)\right\}$, which permutes allelic identities in an ordered manner (i.e., the overall allele ordering from *A* to *B* to *C* is preserved in each pair of alleles), we can take into account the first 9 configurations in the table above:

P[{A,A,B}]+P[{A,A,C}]+P[{B,B,C}]=(321)∑σ∈S2A∫xσ12xσ21p(xσ1,xσ2)dxσ1dxσ2. (17)

In order to include 9 remaining configurations in the table, we need to switch the order of the alleles: {(*A*, *B*), (*A*, *C*), (*B*, *C*)} → {(*B*, *A*), (*C*, *A*), (*C*, *B*)}. But switching the alleles in each pair amounts to replacing $x_{\sigma_{1}}^{2}x_{\sigma_{2}}^{1}$ with $x_{\sigma_{2}}^{2}x_{\sigma_{1}}^{1}=x_{\sigma_{1}}^{1}x_{\sigma_{2}}^{2}$ in Eq 17. Thus we can summarize the entire table by introducing a set *P*(*n*_1_, …, *n*_*k*_) of all distinct permutations of the counts {*n*_1_, …, *n*_*k*_}, which determine the powers to which the allelic frequencies are raised in Eq 17. In our example *P*(2, 1) = {(2, 1), (1, 2)}. Therefore,

P[{2,1}]=(321)∑ν∈P(2,1)∑σ∈S2A∫xσ1ν1xσ2ν2p(xσ1,xσ2)dxσ1dxσ2 (18)

=(321)∑ν∈P(2,1)∑σ∈S2AE[∏i=12xσiνi]. (19)

The above example can be easily generalized to describe the probability $P[\left\{n_{1},\ldots ,n_{k}\right\}]$ of observing arbitrary counts. To do so, we enumerate all *K* alleles, forming a unique ordered list A=(1,…,K). Second, we choose a subset *σ* = (*σ*_1_, …, *σ*_*k*_) of size *k* from A without replacement, so that the allelic order is preserved: *σ*_1_ < … < *σ*_*k*_ (note that no subsets are allowed to contain repeating elements of A). Then $S_{k}A$ can be naturally defined as a set which contains all ordered subsets of A of size *k*. Finally, as before *P*(**n**) is a set of all distinct permutations of allelic counts. Following these steps we have

P[n]=(nn1…nk)∑ν∈P(n)∑σ∈SkAE[∏i=1kxσiνi], (20)

where the expectation is calculated with respect to the steady-state allele distribution, Eq 7.

We can use sampling probability (Eq 20) to compute the distribution of the number of different allelic types *k*:

$$\mathbb{P}\left[k\right]=\sum_{\underset{n_{1}+\ldots +n_{k}=n}{n_{1}\geq \ldots \geq n_{k}}}\mathbb{P}\left[n\right],$$ (21)

where the summation runs over all ordered partitions of *n* into *k* positive integers.

### Generalized sampling formula

As Eq 20 demonstrates, evaluation of sample probabilities requires calculation of moments of allele frequency distributions. This could be done by taking derivatives of the normalization constant Z=B(ϵ)F(ϵ;|ϵ|;β) in Eq 7 with respect to the corresponding components of ***β***:

$$\begin{array}{c} E\left[\prod_{i=1}^{k}x_{i}^{\nu_{i}}\right]=\frac{1}{Z}\prod_{i=1}^{k}{\left(\frac{\partial }{\partial \beta_{i}}\right)}^{\nu_{i}}Z. \end{array}$$ (22)

Then Eq 20 takes the form

P[n]=(nn1…nk)∏i=1kϵ(ni)(Kϵ)(n)∑ν∈P(n)∑σ∈SkAF(ϵ+νσ;Kϵ+n;β)F(ϵ;Kϵ;β), (23)

where ***ν***_*σ*_ is a *K*-dimensional vector whose *σ*_*i*_-th components are *ν*_*i*_ with *i* = 1, …, *k* and all the other components are zero. Here, we have used the fact that differentiating Eq 9 with respect to **z** yields a simple result similar to that known for the regular confluent hypergeometric function:

$$\prod_{i=1}^{k}{\left(\frac{\partial }{\partial z_{i}}\right)}^{n_{i}}\begin{array}{c} F\left(a;b;z\right)=\frac{\prod_{i=1}^{k}{\left(a_{i}\right)}^{\left(n_{i}\right)}}{b^{\left(n\right)}}F\left(a+\sum_{i=1}^{k}n_{i}1_{i};b+n;z\right), \end{array}$$

where $n=\sum_{i=1}^{k}n_{i}$ and (**1**_*i*_)_*j*_ = *δ*_*ij*_. As discussed above, the sum over *σ* extends over all distinct subsets of *k* alleles sampled from *K* uniquely ordered alleles and subject to the *σ*_1_ < … < *σ*_*k*_ constraint. Therefore ***ν***_*σ*_ has *K* − *k* zero and *k* non-zero components which are distributed according to *σ*. The sum over *ν* extends over all distinct permutations of allelic counts which sum up to *n*. Eq 23 is valid for an arbitrary fitness landscape and an arbitrary number of alleles *K*.

### Neutral limit of the sampling formula

When all alleles have the same fitness, the general sampling formula given by Eq 23 should reduce to the Ewens formula for neutral evolutionary dynamics [22, 23]. Indeed, with all *β*_*i*_ set to zero, the generalized hypergeometric function F(a;b;0) (Eq 9) becomes 1. Then for the finite number of alleles *K*

P[n]=NPn!(Kϵ)(n)(Kk)∏i=1kϵ(ni)ni!, (24)

where *N*_*P*_ = |*P*(**n**)| is the total number of distinct permutations of allelic counts. In the limit of an infinite number of alleles *K* → ∞, Eq 24 reduces to Eq 2. Changing variables to allelic histogram counts yields $\prod_{i=1}^{k}n_{i}=\prod_{j=1}^{n}j^{a_{j}}$ and $N_{P}=k!/\prod_{j=1}^{n}a_{j}!$, resulting in

$$\begin{array}{c} P\left[\left(a_{1},\ldots ,a_{n}\right)\right]=\frac{n!}{\prod_{j=1}^{n}a_{j}!j^{a_{j}}}\frac{\theta^{k}}{\theta^{\left(n\right)}}. \end{array}$$ (25)

Eq 25 is a standard form of the Ewens sampling formula [22, 23].

### Sampling formula for a population with two fitness states

As a straightforward generalization of the neutral case, consider a system with *I* alleles of fitness *f*_2_ and *K* − *I* alleles with fitness *f*_1_ > *f*_2_. Thus the fitness landscape consists of two interconnected “planes”. We can assume without loss of generality that alleles 1 through *I* belong to the lower plane and alleles *I* + 1 through *K* belong to the higher plane. Then *γ* = *I*/*K* defines a fraction of nodes on the lower plane and the fitness vector is

$$\begin{array}{c} \beta =(\underset{I}{\underset{︸}{\beta ,\ldots ,\beta }},\underset{K-I}{\underset{︸}{0,\ldots ,0}}), \end{array}$$ (26)

with *I* non-zero entries followed by *K* − *I* zeros, and *β* = −*Ns*. If the first *i* counts come from the lower plane and the other *k* − *i* counts come from the upper plane, we have

$$\begin{array}{c} \nu^{Y}=\left(\underset{I}{\underset{︸}{{\overset{︷}{\nu_{1},\ldots ,\nu_{i}}}^{i},0,\ldots ,0}},\underset{K-I}{\underset{︸}{{\overset{︷}{\nu_{i+1},\ldots ,\nu_{k}}}^{k-i},0,\ldots ,0}}\right), \end{array}$$ (27)

plus all alternative assignments of the first *i* counts within the first *I* entries of ***ν***^*Y*^, and the remaining *k* − *i* counts within the last *K* − *I* entries of ***ν***^*Y*^, such that the original order of the non-zero count entries is not changed. In this case, the generalized hypergeometric function reduces to the confluent hypergeometric function:

F(ϵ+νY;|ϵ|+n;β)=(1F1(γθ+∑m=1iνm;θ+n;β). (28)

Then for finite *K* the sampling probability is given by:

$$\mathbb{P}\left[n\right]=\left(\begin{array}{c} n\\ n_{1}\ldots n_{k} \end{array}\right)\frac{\prod_{i=1}^{k}\epsilon^{\left(n_{i}\right)}}{{\left(K\epsilon \right)}^{\left(n\right)}}\left(\begin{array}{c} K\\ k \end{array}\right)\underset{\nu \in P\left(n\right)}{\sum }\overset{k}{\underset{i=0}{\sum }}\frac{{}_{1}F_{1}\left(\gamma \theta +\sum_{m=1}^{i}\nu_{m};\theta +n;\beta \right)}{{}_{1}F_{1}\left(\gamma \theta ;\theta ;\beta \right)}\frac{\left(\begin{array}{c} I\\ i \end{array}\right)\left(\begin{array}{c} K-I\\ k-i \end{array}\right)}{\left(\begin{array}{c} K\\ k \end{array}\right)}.$$ (29)

Here, the (Ii) and (K-Ik-i) binomial factors are due to assigning non-zero counts to alternative positions within ***ν***^*Y*^, as described above. Taking the infinite allele (*K* → ∞) limit with *γ* fixed, we arrive at

P[n]=n!k!1∏i=1kniθkθ(n)∑ν∈P(n)∑i=0k1F1(γθ+∑m=1iνm;θ+n;β)1F1(γθ;θ;β)(ki)γi(1-γ)k-i. (30)

Thus hypergeometric sampling of Eq 29 reduces to binomial sampling in the infinite-allele limit.

### Sampling formula for a population with multiple fitness states

Let us now generalize the result of the previous section to the case of multiple fitness states: each allele can be assigned a distinct fitness value *f*_*m*_, *m* = 1, …, *M*. In other words, the fitness landscape consists of multiple planes, with *I*_*m*_ = *γ*_*m*_*K* nodes of fitness *f*_*m*_ on the *m*th plane, so that $\sum_{m=1}^{M}\gamma_{m}=1$. Then the sampling probability for finite *K* is given by

$$\mathbb{P}\left[n\right]=\left(\begin{array}{l} n\\ n_{1}\ldots n_{k} \end{array}\right)\frac{\prod_{i=1}^{k}\epsilon^{\left(n_{i}\right)}}{{\left(K\epsilon \right)}^{\left(n\right)}}\left(\begin{array}{c} K\\ k \end{array}\right)\underset{\nu \in P\left(n\right)}{\sum }\underset{Y\in Y\left(I,n\right)}{\sum }\frac{F\left(\gamma \theta +\nu^{Y};\theta +n;\beta \right)}{F\left(\gamma \theta ;\theta ;\beta \right)}\frac{\left(\begin{array}{c} I_{1}\\ i_{1} \end{array}\right)\ldots \left(\begin{array}{c} I_{M}\\ i_{M} \end{array}\right)}{\left(\begin{array}{c} K\\ k \end{array}\right)},$$ (31)

and its infinite allele limit is given by

P[n]=n!k!1∏i=1kniθkθ(n)∑ν∈P(n)∑Y∈Y(n)F(γθ+νY;θ+n;β)F(γθ;θ;β)(ki1…iM)γ1i1…γMiM. (32)

The sums in Eqs 31 and 32 take into account all possible ways of sampling *n* alleles from *M* planes (Fig 1). To explain these sums, let us imagine distributing *n* books over *M* shelves. The books come in *k* indivisible volume sets, and the *i*th set has *ν*_*i*_ identical books in it. We would like to find all book-to-shelf arrangements, keeping in mind that shelves have finite capacities: only *I*_*m*_ books can be placed on the *m*-th shelf. One way to describe any book-to-shelf arrangement is to use an *M*-dimensional vector ***ν***^*Y*^ which records how many books are placed on each shelf. For example, if *M* > *k*, a vector ***ν***^*Y*^ = (*ν*_1_, …, *ν*_*k*_, 0, …, 0) with *M* − *k* zeros following *k* non-zero entries describes placing volume sets on shelves in a particular order: the first volume set goes on the first shelf, the second volume on the second shelf and so on (assuming that the shelves are large enough to accommodate the volume sets), until no more books are left, so that the remaining *M* − *k* shelves remain empty. Permutations of this arrangement, expressed as permutations of ***ν***^*Y*^ vector elements, are also allowed (again, assuming that all the shelves are large enough). We can also put more than one volume set on a single shelf, leading to arrangements such as (*ν*_1_ + *ν*_2_, *ν*_3_, …, *ν*_*k*_, 0, …, 0) with *M* − *k* + 1 zero and *k* − 1 non-zero entries. As before, this arrangement is allowed only if the number of books on each shelf does not exceed shelf capacities. Note that the question of capacity does not arise in the infinite allele limit, since the shelves become effectively infinitely long.

In order to systematically list all the arrangements for volume sets (*ν*_1_, …, *ν*_*k*_), we follow a simple rule: if the *k*th set of *ν*_*k*_ books is placed on the *m*th shelf, the (*k* + 1)th set of *ν*_*k* + 1_ books goes either on the same shelf or on the *m*′th shelf with *m*′ > *m*. Taking elements of (*ν*_1_, …, *ν*_*k*_) one by one and changing the initial shelf (onto which the 1st volume set is placed) and the number of volume sets on each shelf, we can generate a set of all permutations of ***ν***^*Y*^ elements. We shall call this set Y(I,n) since it depends on both the shelf capacities **I** = (*I*_1_, …, *I*_*M*_) and the volume sets **n**. In the limit of infinite shelf capacity the dependence on shelf sizes disappears, and the set of all permutations will be called Y(n). To include all possible arrangements, we need to perform the book-placing procedure for each distinct permutation of **n**.

Now, if we replace shelves with fitness planes and volume sets with allelic counts, we obtain an algorithm for generating all allowed placements of allelic counts on fitness planes. The non-negative indices *i*_1_, …, *i*_*M*_ in Eqs 31 and 32 represent the number of volume sets (allelic counts) on each shelf (fitness plane). The distribution of alleles among fitness planes of finite capacity is illustrated in Fig 1A for *M* = 3 and a vector of allelic counts ***ν*** = (4, 1, 2); the infinite-plane case is shown in Fig 1B.

Next, let us consider the monomorphic limit of Eq 32. It can be shown that

$$\begin{array}{c} F\left(\theta \gamma ;\theta ;\beta \right)\underset{\theta \to 0}{\to }\sum_{m=1}^{M}\gamma_{m}e^{\beta_{m}}, \end{array}$$ (33)

leading to

$$\begin{array}{c} \begin{array}{c} P[\{n\}]=1+O(\theta ),\\ P[\left\{n_{1},\ldots ,n_{k}\right\}]=O(\theta^{k-1}). \end{array} \end{array}$$ (34)

Therefore, as expected, the P[{n}] (*k* = 1) term dominates in the monomorphic limit.

By construction, Eq 32 reduces to the neutral limit, Eq 2, when all fitness values are the same. In addition, the neutral limit is reproduced in the strongly polymorphic limit

$$\begin{array}{c} F\left(\gamma \theta +\nu_{Y};\theta +n;\beta \right)\underset{\theta \to \infty }{\to }F\left(\gamma \theta ;\theta ;\beta \right), \end{array}$$ (35)

and Eq 32 reduces to the neutral result. This is expected since selection effects become vanishingly small in this regime.

### Efficient evaluation of sampling probabilities

To evaluate sampling probabilities, we need to compute F(a,b,z) (Eq 9) efficiently. The calculation of F(a,b,z) is performed by filling a square matrix with the partial Bell polynomials *B*_*n*, *k*_, from which complete Bell polynomials can be calculated from the rows as $B_{n}=\sum_{k=1}^{n}B_{n,k}$. We use the following convolution identity: (x♢y)n=∑j=1n-1(nj)xjyn-j. Note that the identity is commutative, i.e. ${\left(x♢y\right)}_{n}={\left(y♢x\right)}_{n}$, and that the summation limits are such that the convolution of two vectors with nonzero elements will always have a zero as its first element. Let $x^{k♢}$ denote the vector that results when **x** is convolved with itself *k* times. The convolution matrix *C* is lower triangular and has the vector **x** = (*x*_1_, …, *x*_*n*_)^*T*^ as its leftmost column, $x^{2♢}$ as the second leftmost, etc. Partial Bell polynomials can then be calculated as:

$$\begin{array}{c} B_{n,k}\left(x_{1},\ldots ,x_{n-k+1}\right)=\frac{{\left(x^{k♢}\right)}_{n}}{k!}=\frac{C_{n,k}}{k!}. \end{array}$$ (36)

The matrix elements *C*_*n*, *k*_ can be calculated starting from the top of the matrix, left-to-right within each row. The sum in Eq 9 runs over complete Bell polynomials in ascending order, so that convergence can be checked after the completion of each row. We specify a relative precision, e.g. $\tilde{\epsilon }=10^{-12}$, and terminate the computation of F once the contribution of the current term *j* is small enough compared to the partial sum from 0 to *j* − 1: $|F_{j}/F_{\text{partial}}|<\tilde{\epsilon }$.
